# A comparison of approaches for adjudicating outcomes in clinical trials

**DOI:** 10.1186/s13063-017-1995-3

**Published:** 2017-06-08

**Authors:** Brennan C. Kahan, Brian Feagan, Vipul Jairath

**Affiliations:** 1 Pragmatic Clinical Trials Unit, Queen Mary University of London, 58 Turner St, London, E1 2AB UK; 2 Robarts Clinical Trials, London, ON Canada; 3 Department of Medicine, Western University, London, ON Canada; 4 Department of Epidemiology and Biostatistics, Western University, London, ON Canada

**Keywords:** Randomized controlled trial, Outcome adjudication, Outcome assessment, Endpoint adjudication committee, Endpoint review committee, Misclassification, Central assessor

## Abstract

**Background:**

Incorrect classification of outcomes in clinical trials can lead to biased estimates of treatment effect and reduced power. Ensuring appropriate adjudication methods to minimize outcome misclassification is therefore essential. While there are many reported adjudication approaches, there is little consensus over which approach is best.

**Methods:**

Under the assumption of non-differential assessment (i.e. that misclassification rates are the same in each treatment arm, as would typically be the case when outcome assessors are blinded), we use simulation and theoretical results to address four different questions about outcome adjudication: (a) How many assessors should be used? (b) When is it better to use onsite or central assessment? (c) Should central assessors adjudicate all outcomes, or only suspected events? (d) Should central assessment with multiple assessors be done independently or through group consensus?

**Results:**

No one adjudication approach performs optimally in all settings. The optimal approach depends on the misclassification rates of site and central assessors, and the correlation between assessors. We found: (a) there will generally be little incremental benefit to using more than three assessors and, for outcomes with very high correlation between assessors, using one assessor is sufficient; (b) when choosing between site and central assessors, the assessor with the smallest misclassification rate should be chosen; when these rates are unknown, a combination of one site assessor and two central assessors will provide good results across a range of scenarios; (c) having central assessors adjudicate only suspected events will typically increase bias, and should be avoided, unless the threshold for sending outcomes for central assessment is extremely low; (d) central assessors can adjudicate either independently or in a group, and the preferred option should be dictated by whichever is expected to have the lowest misclassification rate.

**Conclusions:**

Outcome adjudication is of critical importance to ensure validity of trial results, although no one approach is optimal across all settings. Investigators should choose the best strategy based on the specific characteristics of their trial. Regardless of the adjudication strategy chosen, assessors should be qualified and receive appropriate training.

## Background

Many randomized controlled trials involve binary outcomes where either an event occurred or it did not (e.g. myocardial infarction, disease progression, patient response). In some situations it is self-evident whether an event occurred (e.g. mortality). However, in most situations it is less clear and may involve some subjectivity; thus, the outcome may need to be adjudicated to determine whether an event occurred. Appropriate adjudication of outcomes is of critical importance to the validity of trial results, as poor adjudication can lead to bias and a loss of power [1]. However there is little empirical evidence to help trial organizers select the best adjudication approach in various settings.

For example, clinical trials of ulcerative colitis often use an endoscopic video to determine whether an ulcer or a degree of inflammatory change is present or absent. There are several potential adjudication approaches in this setting. The outcome could be adjudicated either by a site assessor, who is directly involved in the patient’s care, and may be influenced by knowledge of the patient’s symptoms and their clinical history, or by a central assessor, who would only have access to the endoscopic video, and is blinded to all other clinical information. The number of adjudicators could be varied. The type of outcomes sent to the central assessors could vary (e.g. all outcomes vs. only suspected events). Different methods of adjudication could be used (for example, independent adjudication vs. group adjudication). There is currently little evidence to inform these choices.

Assuming that there is a ‘true’ but unknown outcome, there are four possible results after adjudication (Table 1); true-positives and true-negatives (where the assessor correctly judges that the event did or did not occur) and false-positives and false-negatives (where the assessor incorrectly judges that the event did or did not occur).

**Table 1:** Possible scenarios after adjudication

		Adjudicated outcome	
		No event	Event
True outcome	No event	True-negative	False-positive
	Event	False-negative	True-positive

False-positives and false-negatives are forms of misclassification [1]. Assuming that the misclassification is non-differential (i.e. that there is the same probability of a false-negative or false-positive in each treatment arm, as would generally be the case when assessors are blinded to treatment arm), misclassification will lead to downward bias in the estimated treatment effect (i.e. the estimated treatment effect will be closer to the null than the true treatment effect), provided the true treatment effect is non-null [1]. When the treatment effect is null, misclassification will not lead to bias.

The extent of the bias under a non-null treatment effect depends on the rate of misclassification; the larger the rate of misclassification, the higher the degree of bias. For example, if the treatment effect was a difference in percentage points, the estimated treatment effect in the presence of misclassification will be biased by a factor of:

1 $$ 1-\varphi -\theta $$

where *φ* is the false-positive rate (the proportion of non-events that were incorrectly classified as events) and *θ* is the false-negative rate (the proportion of events that were incorrectly classified as non-events) [2]. For example, assuming that the true difference in percentage points is 20%, and the false-positive and false-negative rates are both 5%, the estimated treatment effect would be 18% (i.e. it would be biased downwards by 10%). If, however, the false-positive and false-negative rates were both 15%, the estimated treatment effect would be 14% (a downward bias of 30%). Misclassification will also lead to a reduction in power, both because of the downward bias in the estimated treatment effect and because misclassification affects the variance of the estimated treatment effect [1].

Given the adverse consequences of misclassification of outcomes, reducing the misclassification rate is of critical importance to ensure the validity of results in randomized controlled trials. A number of case studies have compared results from different adjudication approaches in specific trials [3–15]; however, little attention has been paid to the statistical properties of the different approaches. In this paper, we address four main questions related to outcome adjudication: (a) How many assessors should we use? (b) Should we use site or central assessors to adjudicate outcomes? (c) Should central assessors adjudicate all outcomes, or only suspected events? (d) If we use central assessment with multiple assessors, should adjudication be done by each assessor independently, or as a group consensus (e.g. in an endpoint review committee)?

## Methods

In this paper, we focus on the assessment approach for binary outcomes, and assume that there is non-differential assessment (i.e. the false-positive and false-negative rates are the same in all treatment arms), as would typically be the case in a double-blind trial or in trials where all assessors are blinded to treatment allocation.

We begin by describing a relevant example from clinical trials in ulcerative colitis. We then discuss the notation to be used, followed by an overview of the statistical properties of several different adjudication approaches. We then describe a simulation study comparing the different adjudication approaches. Key terminology used in this paper is listed in Table 2.

**Table 2:** Terminology and definitions

Term	Definition
Site assessor	An assessor based at a local site or hospital, who may or may not be involved in the patient’s care
Central assessor	An assessor who is based centrally, not at any local site or hospital, and is not involved in the patient’s care
Adjudication	The process of classifying the outcome as an event or a non-event
Misclassification	Incorrectly classifying the outcome
False-positive	Incorrectly classifying the outcome as an event when the true outcome is a non-event (the false-positive rate is denoted *φ*)
False-negative	Incorrectly classifying the outcome as a non-event when the true outcome is an event (the false-negative rate is denoted *θ*)

### Clinical trials in ulcerative colitis

Ulcerative colitis is a chronic, relapsing disorder of the colon resulting from an excessive immune response against environmental antigens, although the aetiology is unknown. Patients typically present with symptoms of abdominal pain, bleeding and diarrhoea. Most clinical trials for ulcerative colitis require adjudication of endoscopy results (an examination of the patient’s colon using a fibre-optic camera) to determine whether there is evidence of disease activity (e.g. ulceration, inflammation, bleeding). The results of the endoscopy are used to inform the outcome of clinical remission (yes vs. no), either alone or in conjunction with other information, such as patient-reported symptoms and a global physician assessment [16].

The results from the endoscopy could be adjudicated based on a number of different approaches. For example, the results could be adjudicated by the local onsite clinician who performs the endoscopy (site assessor), or by a clinician not based at the site who adjudicates the outcome based on a review of the endoscopic video (central assessor).

Furthermore, a central assessor, if used, could adjudicate either all patients or only a subset of patients. For example, one adjudication strategy could be to have a site assessor perform an initial adjudication, and then have a central assessor to adjudicate suspected events only. Additionally, under central adjudication, any number of assessors could be used. For example, instead of using only one assessor, three assessors could be used. This would typically involve two assessors performing an initial adjudication, and if they disagree, a third assessor acting as a tiebreak. This approach could be generalized to any odd number of assessors, e.g. 3, 5, 7, 9, etc. An additional challenge in using central adjudication is whether to only include the assessments made by the central assessor(s), or to use a hybrid approach, which also includes the assessment made by the site assessor.

Finally, under central adjudication with multiple assessors, the adjudication could be done either, independently by each assessor, or all together, as a group (if the practicalities of the trial allow for this). Under the independent assessment approach, each assessor performs the adjudication independently, without knowledge of results from the other assessors; the final classification is then based on a ‘majority rules’ approach. For example, with three assessors, the outcome is classified as an event if the majority of assessors (two or more) adjudicate an event (and vice versa for no event). Conversely, the group approach would typically involve an endpoint review committee, in which all assessors meet and conduct the adjudication together.

In the following sections, we outline some of the statistical considerations of these differing adjudication approaches. We then discuss some considerations for choosing an adjudication approach in ulcerative colitis clinical trials later in the paper.

### Notation

Before considering any of the listed adjudication scenarios in detail, we outline some notation to be used. Let *Y*_*i*_denote the true outcome for the *i*th patient, with *Y*_*i*_ = 1 indicating that an event occurred and *Y*_*i*_ = 0 indicating that no event occurred. Assume that there are *j* different assessors; then, let *Y*_ij_^*^ denote the adjudicated outcome for the *i*th patient from the *j*th assessor: *Y*_ij_^*^ = 1 means that the assessor has adjudicated an event, and *Y*_ij_^*^ = 0 means that the assessor has adjudicated no event. Finally, let *Y*_i_^*^ denote the final adjudicated outcome for the *i*th patient (which is calculated based on the individual *Y*_ij_^*^s).

Then

$$ P\left({Y}_i^{*}=1\Big|{Y}_i=0\right)=\varphi $$

denotes the false-positive rate (the probability of a non-event being incorrectly classified as an event), and

$$ P\left({Y}_i^{*}=0\Big|{Y}_i=1\right)=\theta $$

denotes the false-negative rate (the probability of an event being incorrectly classified as a non-event).

Let

$$ \beta ={p}_1-{p}_0 $$

where *β* is the true treatment effect, and *p*_1_ and *p*_0_ are the true probabilities of an event in the intervention and control arm respectively. Therefore, *β* represents the difference in proportions between treatment arms. The estimated treatment effect, $\widehat{\beta}$, is

$$ \widehat{\beta}={\widehat{p}}_1-{\widehat{p}}_0 $$

where ${\widehat{p}}_1$ and ${\widehat{p}}_0$ are the estimated event rates in the intervention and control arms, respectively.

In the presence of misclassification,

$$ \widehat{\beta} = \beta \left(1 - \varphi - \theta \right) $$

indicating that the estimated treatment effect will always be closer to zero (indicating no difference between treatment groups) than the true difference, and that if *β* = 0 (indicating no difference between treatment groups) then $\widehat{\beta}=\beta$, and our estimate will be unbiased.

Finally, we define the infraclass correlation (ICC) as

$$ \frac{P\left({Y}_{i j}^{*}={Y}_{i j\hbox{'}}^{*}\right)- P\left({Y}_{i j}^{*}={Y}_{i\hbox{'} j\hbox{'}}^{*}\right)}{1- P\left({Y}_{i j}^{*}={Y}_{i\hbox{'} j\hbox{'}}^{*}\right)},\ \mathrm{f}\mathrm{o}\mathrm{r}\  i\ne {i}^{\hbox{'}},\ \mathrm{and}\  j\ne j\hbox{'} $$

where *P*(*Y*_ij_^*^ = *Y*_*ij*′_^*^) denotes the probability that two different assessors make the same classification for the same patient, and *P*(*Y*_ij_^*^ = *Y*_i′j′_^*^) denotes the probability that two different assessors make the same classification for different patients [17]. The ICC measures how similar classifications from different assessors are; a value of zero indicates that assessors are no more likely than chance to make the same assessment, whereas a value of one indicates that assessors always make the same assessment. This measure is equivalent to the kappa index [17, 18], which is used to quantify the interrater agreement between different assessors.

### Overview of different scenarios

#### How many assessors should we use?

We begin with the simplest question – if we were using only central assessors, how many assessors should we use? For the moment, we assume that adjudication will be done independently by each assessor, and that the final classification will be based on a ‘majority rules’ approach. This approach requires an odd number of assessors (though could initially be undertaken by fewer assessors, with the final assessor only called on, if required, to break a tie).

Adjudication requires time, effort, and other resources [9, 14, 19]; each additional assessor will come at a cost. In some trials, this could be substantial [14]. Therefore, the number of assessors should only be increased when the benefit outweighs the costs.

The potential advantage of increasing the number of assessors can be seen in the following example. Assume that all assessors have a false-positive rate of 10% (i.e. 10% of the time they will falsely classify a non-event as an event). Furthermore, let us assume that results from each assessor are completely independent (i.e. that ICC = 0, and that assessors are no more likely than chance to make the same classification). Then, using only one assessor will lead to an overall false-positive rate of 10%. However, with three assessors, a false-positive will only occur if two or more assessors get the classification wrong. Therefore, with three assessors, the overall false-positive rate can be calculated as:

$$ P\left({\displaystyle \sum_{j=1}^3}{Y}_{i j}^{*}\ge 2\ \Big|\ {Y}_i=0\right) = \left(\begin{array}{c}\hfill 3\hfill \\ {}\hfill 2\hfill \end{array}\right){0.1}^2{0.9}^1+\left(\begin{array}{c}\hfill 3\hfill \\ {}\hfill 3\hfill \end{array}\right){0.1}^3{0.9}^0=0.028 $$

i.e. using three assessors can reduce the overall false-positive rate from 10% to only 2.8%, which could substantially reduce bias and increase power.

However, if we make the alternate assumption that ICC = 1 (i.e. that assessors will always make the same classification), then all three assessors would essentially function as one. In this situation, increasing the number of assessors from one to three has no impact, and the overall false-positive rate will be unchanged at 10%. Thus, increasing the number of assessors can reduce misclassification, thereby reducing bias and increasing power, but the extent of this benefit will depend on the correlation between different assessors; the higher the correlation, the less incremental benefit of adding extra assessors.

#### Should we use site or central assessors?

Assuming that adjudication could be performed by either site assessors or central assessors (and that both parties are blind to treatment allocation), a natural question is which type of assessor to use. Central assessment typically requires additional resources compared with site assessment. For example, information needs to be collated and sent to the central assessors, who then need to take time out to perform the adjudication, often in a time-sensitive manner. Furthermore, specialized equipment may sometimes be required to record the event. Conversely, site assessment can be done on the spot, and is typically (though not always) done routinely. Therefore, using central assessment usually carries a resource burden, and should only be used when the benefit of doing so outweighs the costs.

In practice, central assessment will benefit the trial when central assessors have lower misclassification rates than site assessors. Therefore, when central assessors are expected to be better at classifying outcomes, central assessment should be the preferred option. Conversely, when site assessors are expected to have better classification rates, they should be the preferred option. When the classification rates are expected to be approximately the same between site and central assessors, then either option could be used.

In practice, however, the comparative misclassification rates of site and central assessors are often unknown and may be difficult to predict. In this situation, it is not clear which adjudication approach to use. We could then guess at which type of assessor is likely to be better, based on the specific trial characteristics. For example, for outcomes that are easier to adjudicate and do not require much experience or specific training (e.g. whether a patient is able to walk 10 m across a room unaided), site and central assessors are likely to be comparable, and so using a site assessor to reduce resource burden makes sense. Conversely, for more challenging outcomes, which require experience and specific training (e.g. occurrence of myocardial infarction), using central assessors may be the best approach, as it will be easier to ensure that a small number of central assessors are sufficiently experienced and have received appropriate and consistent training than to ensure the same for a large number of site assessors.

An alternative option, when the comparative misclassification rates of site and central assessors are unknown, is to use a combination approach to adjudication; instead of using either site or central assessors, both could be used. For example, the independent adjudication of both the site and a central assessor could be used, with a second central assessor (a third overall assessor) used if a tiebreak is required.

Under the assumption of independence between assessors (i.e. ICC = 0), we can derive the overall false-positive rate for the approach of using one site assessor and two central assessors as follows. Let *φ*_SA_ and *φ*_CA_ denote the false-positive rates of the site and central assessors respectively, and *θ*_SA_ and *θ*_CA_ denote their false-negative rates. Then, the false-positive rate for the approach of using one site assessor and two central assessors (denoted *φ*_1SA + 2CA_) is:

$$ {\varphi}_{1\mathrm{SA}+2\mathrm{CA}}=2{\varphi}_{\mathrm{SA}}{\varphi}_{\mathrm{CA}}\left(1-{\varphi}_{\mathrm{CA}}\right)+{\varphi_{\mathrm{CA}}}^2\left(1-{\varphi}_{\mathrm{SA}}\right)+{\varphi}_{\mathrm{SA}}{\varphi_{\mathrm{CA}}}^2 $$

**Fig. 1:**
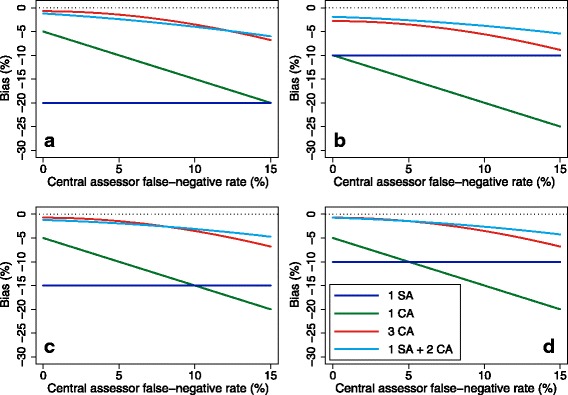
Performance of combination approach, with one site assessor and two central assessors, compared with other strategies. CA, central assessor; SA, site assessor. **a** Site assessor, 10% false-positive rate and 10% false-negative rate; central assessor, 5% false-positive rate. **b** Site assessor, 5% false-positive rate and 5% false-negative rate; central assessor, 10% false-positive rate. **c** Site assessor, 10% false-positive rate and 5% false-negative rate; central assessor, 5% false-positive rate. **d** Site assessor, 5% false-positive rate and 5% false-negative rate; central assessor, 5% false-positive rate

The false-negative rate is derived similarly. We can see in Fig. 1 that this approach generally compares favourably with other potential approaches (i.e. either one site or one central assessor, or three central assessors) across a range of scenarios.

#### Should central assessors adjudicate all outcomes, or only suspected events?

**Fig. 2:**
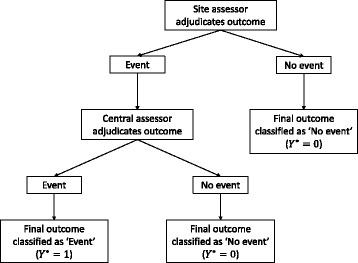
Two-stage adjudication approach. CA, central assessor; SA, site assessor

A common adjudication strategy is for site assessors to perform an initial adjudication of all outcomes, and then for central assessors to re-adjudicate suspected events. This strategy is outlined in Fig. 2; if the site assessor adjudicates no event, the outcome is classified as no event. If the site assessor adjudicates an event, the outcome is passed on to the central assessor(s), who will re-adjudicate it, with their decision being final. Under this approach, the outcome will be classified as an event if *both* the site and central assessors adjudicate an event, and it will be classified as no event if *either* the site or central assessors adjudicate no event. We refer to this adjudication approach as a *two-stage approach* in this paper.

The two-stage approach is likely to be used in situations where it is believed that central assessors have better classification rates than site assessors, but central assessors come with a cost or resource burden, and so it is more economical to only have central assessors adjudicate a subset of outcomes in order to reduce costs. Therefore, a relevant question is: Under which circumstances is it acceptable to have central assessors only assess suspected events, rather than all outcomes? In the remainder of this section, we compare the two approaches (the two-stage approach vs. having central assessors adjudicate all outcomes).

Under the assumption of independence between site and central assessors (i.e. ICC = 0), we can derive the overall false-positive and false-negative rates under the two-stage approach as follows. Let *φ*_SA_ and *φ*_CA_ denote the false-positive rates of the site and central assessors respectively, and *θ*_SA_ and *θ*_CA_ denote their false-negative rates. Then, the false-positive and false-negative rates for the two-stage approach (denoted *φ*_2stage_ and *θ*_2stage_, respectively) can be calculated as

$$ {\varphi}_{2\mathrm{stage}}={\varphi}_{\mathrm{SA}}{\varphi}_{\mathrm{CA}} $$

and

$$ {\theta}_{2\mathrm{stage}}={\theta}_{\mathrm{SA}}+{\theta}_{\mathrm{CA}}-{\theta}_{\mathrm{SA}}{\theta}_{\mathrm{CA}} $$

Because *φ*_SA_, *φ*_CA_, *θ*_SA_ and *θ*_CA_ are all between 0 and 1, this indicates that

$$ {\varphi}_{2\mathrm{stage}}\le {\varphi}_{\mathrm{SA}},\ {\varphi}_{\mathrm{CA}} $$

and

$$ {\theta}_{2\mathrm{stage}}\ge {\theta}_{\mathrm{SA}},\ {\theta}_{\mathrm{CA}} $$

That is, the two-stage approach will always have a false-positive rate that is less than or equal to that of a site or central assessor alone; however, it will always have a false-negative rate that is equal or higher. Therefore, the two-stage approach will only be as good as or better than using a central assessor to adjudicate all outcomes when the reduction in the false-positive rate is equal to or greater than the increase in the false-negative rate, i.e. when

$$ {\varphi}_{\mathrm{CA}}-{\varphi}_{2\mathrm{stage}}\ge {\theta}_{2\mathrm{stage}}-{\theta}_{\mathrm{CA}} $$

This expression reduces to

$$ {\varphi}_{\mathrm{CA}}\left(1-{\varphi}_{\mathrm{SA}}\right)\ge {\theta}_{\mathrm{SA}}\left(1-{\theta}_{\mathrm{CA}}\right) $$

This expression relies on several items; however, we can see that a primary factors that will influence whether the two-stage approach is useful is the false-negative rate of the site assessors; when this is very small, using the two-stage approach is likely to be beneficial. However, the assumption that site assessors will have very low false-negative rates is contradictory to the assumption behind the two-stage approach, which is that the site assessor’s misclassification rates are high enough that suspected events require further adjudication by central assessors to correct any mistakes the site assessor has made.

This highlights the contradiction in logic behind the two-stage approach; that site assessors cannot be trusted to determine whether an event occurred, but can be trusted to determine whether an event did not occur. This approach assumes that correctly classifying events is more important than correctly classifying non-events, which is not the case; as seen in formula (1), misclassification of both events and non-events has the same effect on bias.

**Fig. 3:**
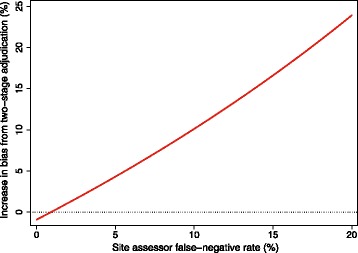
Increase in bias from two-stage adjudication approach compared with central assessment. This graph shows the increase in bias from using a two-stage approach (where central assessors adjudicate only suspected events) compared with having one central assessor adjudicate all events and is based on a scenario where the central assessor has better classification rates than the site assessor in general; the false-positive and false-negative rates for the central assessor are 1%, and the false-positive rate for the site assessor is 10%. The false-negative rate for the site assessor is shown on the *x*-axis

The bias of the two-stage approach (relative to having a central assessor adjudicate all events) is shown in Fig. 3 for different values of the site assessor’s false-negative rate. This graph reflects the situation where the central assessor has lower rates of misclassification than the site assessor (as would typically be assumed when this approach is used). From the graph, we can see that the two-stage approach is only as good as having a central assessor adjudicate all outcomes when the site assessor’s false-negative rate is close to zero.

From Fig. 3, we can see that, rather than have site assessors pass on outcomes they think are events to central assessors, a better approach would be to have them pass on all outcomes apart from those they can rule out as definitive non-events (i.e. they would still pass on outcomes they think are non-events, provided there is any possibility that it could be an event). By lowering the threshold at which outcomes are sent to the central assessors, the site assessor’s false-negative rate is minimized, allowing central assessors to adjudicate relevant outcomes (i.e. all outcomes except those which are definitely not events) while still reducing the overall burden compared with having the central assessors adjudicate all outcomes. However, this approach does rely on site assessors being definitely able to identify outcomes that are not events; if this is unlikely to be the case, this modified approach is unlikely to be useful.

#### Independent vs. group adjudication for central assessors

If multiple central assessors are used for adjudication, they could either conduct the adjudication independently (i.e. each could adjudicate alone, with no knowledge of results from other assessors), or they could perform the adjudication in a group if trial practicalities permit (e.g. as part of an endpoint review committee), where each patient is discussed, and the final classification is decided together. Under group adjudication, the outcome could either be decided by a vote (with a ‘majority rules’ approach), by consensus, with disagreements resolved by discussion (although in practice, achieving consensus between assessors for all patients may be impossible), or through another method (e.g. a Delphi process [20, 21]).

Group adjudication may require additional resources compared with independent adjudication, for example, additional travel, time, or communication costs. Therefore, it is useful to know when we might expect an improvement in classification under the group approach, in order to determine whether the additional resource and logistical requirements are worthwhile.

Depending on the dynamics of the group, we might reasonably expect group adjudication to either increase or decrease the classification rates compared with independent adjudication and, potentially, to increase the correlation between assessors. For example, there may be one dominant voice or an opinion leader in the group that the other assessors follow; this would have the effect of increasing correlation between individuals (as the group would effectively act as one voice). Furthermore, if the dominant voice belongs to an assessor with poor classification rates, this could increase the overall misclassification rate compared with independent adjudication. Conversely, if the dynamic is one of collaboration and engagement, a discussion of each patient may allow the group to arrive at the correct classification more often than if the assessors had done the adjudication alone.

In practice, group adjudication may be preferred if it is expected that this would lead to better classification rates than achieved for independent adjudication. However, this must be balanced against the potential increase in correlation between assessors from group adjudication; as seen earlier, the higher the correlation, the less benefit there is to using more than one assessor. Therefore, if group adjudication increases the classification rate by a small margin, but leads to a substantial increase in the correlation, it may be that using independent adjudication actually leads to slightly better classification rates overall.

### Simulation study

We conducted a simulation study to compare the different adjudication approaches across these scenarios. As denoted, *Y*_*i*_ represents the true outcome for the *i*th patient, with *Y*_*i*_ = 1 indicating that an event occurred, and *Y*_*i*_ = 0 indicating that no event occurred. *Y*_ij_^*^ represents the adjudicated outcome for the *i*th patient from the *j*th assessor. *Y*_ij_^*^ = 1 indicates that the assessor has adjudicated an event, and *Y*_ij_^*^ = 0 indicates that the assessor has adjudicated no event. Finally, *Y*_i_^*^ represents the final adjudicated outcome for the *i*th patient (which is calculated based on the individual values of *Y*_ij_^*^).

We generated the true patient outcomes from the following model:

$$ P\left({Y}_i=1\right)=\alpha +\beta {X}_i $$

where *α* is the probability of an event in the control arm, *X*_*i*_ represents the treatment group to which the *i*th patient was allocated (*X*_*i*_ = 1 represents the intervention group, and *X*_*i*_ = 0 represents the control group), and, as before, *β* is the difference in proportions between the intervention and control arm (representing the treatment effect).

For all simulation scenarios we set *n* = 1000 (500 patients in each of the two treatment arms), and *α* = 0.5 (denoting a 50% event rate in the control). We set *β* = −0.088 (indicating an event rate of 41.2% in the intervention arm, representing a difference in percentage points of −8.8%). We chose these values of *α* and *β* to give 80% power under perfect adjudication.

We generated the adjudicated outcomes from central assessors using a beta-binomial distribution with a specified ICC (representing the degree of correlation or agreement between different central assessors), and with a specified false-positive and false-negative rate. For scenarios that included a site assessor, we generated adjudicated outcomes from the site assessor based on the same beta-binomial model as for the central assessor, but using the specified false-positive and false-negative rates for the site assessor. This means that the correlation between site and central assessors will be less than the correlation between different central assessors, owing to the differences in the false-positive and false-negative rates.

Details of how we calculated an overall adjudicated outcome (*Y*_i_^*^) for each scenario are provided next. Once we calculated *Y*_i_^*^ for each patient, we used these adjudicated outcomes to estimate $\widehat{\beta}$. This was done using a generalised linear model with a binomial family and an identity link; we used *Y*_i_^*^ as the outcome, and the treatment arm (*X*_*i*_) as the only covariate.

Then, for each different adjudication approach, we estimated the following:

- Percentage bias in $\widehat{\beta}$
- Power

The percentage bias in $\widehat{\beta}$ was calculated as

$$ \mathrm{Percentage}\ \mathrm{bias}\ \mathrm{in}\kern0.5em \widehat{\beta} = \frac{\left(\widehat{\beta}-\beta \right)}{\beta} $$

Power was defined as the percentage of replications for which *p* < 0.05.

For all simulation scenarios, we used 10,000 replications. We provide further details on each specific simulation scenario next.

#### How many assessors should we use?

We evaluated using either one, three, five, or seven central assessors. With multiple assessors, the final classification of the outcome was based on the adjudication of the majority of assessors (e.g. with three assessors, the final classification was ‘event’ if two or more assessors adjudicated ‘event’, and vice versa). With only one assessor, the final classification of the outcome was set to the one assessor’s adjudicated outcome. Formally:

$$ {Y}_i^{*}=\left\{\begin{array}{c}\hfill 0\kern0.5em \mathrm{if}\ {\displaystyle \sum_{j=1}^m}{Y}_{i j}^{*}<\frac{m+1}{2}\hfill \\ {}\hfill 1\kern0.5em \mathrm{if}\ {\displaystyle \sum_{j=1}^m}{Y}_{i j}^{*}\ge \frac{m+1}{2}\hfill \end{array}\right. $$

where *m* represents the total number of assessors used.

We varied the correlation between assessors (ICC) and the assessors’ false-positive and false-negative rates (*φ* and *θ*). We set the false-positive and false-negative rate to be equal for each assessor, e.g. if *φ* was 5% then θ was also set to 5%. We varied the ICC between 0.10, 0.25, 0.50, 0.75 and 0.90, and varied *φ* and *θ* between 5%, 10%, 15% and 20%. This led to 5 × 4 = 20 total scenarios.

#### Should we use site or central assessors?

We evaluated four different approaches of adjudication:

- One site assessor
- One central assessor
- Three central assessors
- One site assessor and two central assessors

For the approach with three central assessors, the final classification of the outcome was calculated as before (i.e. based on the majority of adjudications). For the approaches with one site assessor or one central assessor, the final classification was based on the assessor’s adjudicated outcome. For the approach with one site assessor and two central assessors, the final classification of the outcome was based on the adjudication of the majority of assessors (the same method used for three central assessors), regardless of whether they were site or central assessors. For example, the final classification was an ‘event’ if two or more assessors adjudicated ‘event’, regardless of whether these were the two central assessors, or one central assessor and one site assessor.

We varied the ICC and the false-positive and false-negative rates for the different assessors. We varied the ICC between 0.10, 0.25, 0.50, 0.75 and 0.90. We used three different scenarios for the false-positive and false-negative rates of the assessors:

- Scenario 1: central assessors have lower false-positive and false-negative rates:
  - ○ Site assessor: *φ*_SA_ = 20%, *θ*_SA_ = 20%
  - ○ Central assessor: *φ*_CA_ = 10%, *θ*_CA_ = 10%
- Scenario 2: site assessors have lower false-positive and false-negative rates:
  - ○ Site assessor: *φ*_SA_ = 10%, *θ*_SA_ = 10%
  - ○ Central assessor: *φ*_CA_ = 20%, *θ*_CA_ = 20%
- Scenario 3: site assessors have lower false-negative rates, central assessors have lower false-positive rates:
  - ○ Site assessor: *φ*_SA_ = 20%, *θ*_SA_ = 10%
  - ○ Central assessor: *φ*_CA_ = 10%, *θ*_CA_ = 20%

This led to 5 × 3 = 15 total scenarios.

#### Should central assessors adjudicate all outcomes, or only suspected events?

We evaluated three different approaches to adjudication:

- One site assessor
- One central assessor
- Two-stage approach

We simulated a scenario where the central assessor generally had better classification rates than the site assessor (as would generally be the assumption if a two-stage adjudication approach were adopted).

For the central assessor, we set both the false-positive and false-negative rates to 5% (i.e. *φ*_CA_ = *θ*_CA_ = 5%); for the site assessor, we set the false-positive rate to 10% (*φ*_SA_ = 10%). We then varied the false-negative rate for the site assessor (*θ*_SA_) between 5%, 10%, 15% and 20%. We varied the ICC between 0.10, 0.50 and 0.90 (note that this ICC is conditional having the same misclassification rate, and so is not exact between site and central assessors).

This led to 4 × 3 = 12 total scenarios.

#### Independent vs. group adjudication for central assessors

We explored the scenario where multiple central assessors will adjudicate the outcome, and evaluated two different approaches:

- Central assessors adjudicate the outcome independently (independent adjudication).
- Central assessors adjudicate the outcome together in a group (group adjudication).

For each method, we used three central assessors. Final classification of the outcome for independent adjudication was based on the majority. For group adjudication, we assumed an approach where assessors would discuss each outcome before voting, with the final classification being based on the majority.

We assumed that group adjudication could change the classification rate compared with independent adjudication (either increase or decrease it), and could increase the correlation between different assessors.

We set the false-positive and false-negative rates to 20%, and the ICC to 0.50 for independent adjudication. Under group adjudication, we varied the change in the false-positive and false-negative rates between −2%, −1%, 0%, 1% and 2%. A change of −2% indicates that using group adjudication led to a decrease of 2% for both the false-positive and false-negative rate compared with independent adjudication (i.e. the rates were reduced from 20% to 18%). We varied the change in ICC under group adjudication between 0, 0.1, 0.2, 0.3 and 0.4. A change of 0.4 indicates that using group adjudication led to an increase in the ICC of 0.4 compared with independent adjudication (i.e. the ICC was increased from 0.50 to 0.90).

This led to 5 × 5 = 25 total scenarios.

## Results

### How many assessors should we use?

**Fig. 4:**
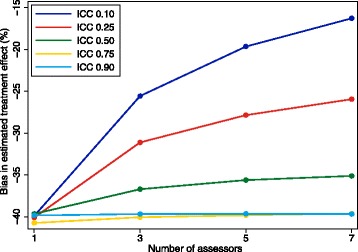
Bias with different numbers of assessors. This figure shows the association between increasing the number of assessors and reducing the bias in the estimated treatment effect. The intraclass correlation coefficient (ICC) represents the degree of correlation between different assessors, with a value of zero indicating no more agreement between assessors than would be expected by chance, and a value of one indicating perfect agreement between assessors. The false-positive and false-negative rate were both 20%

**Fig. 5:**
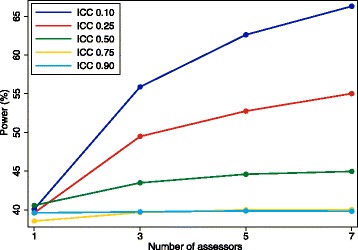
Power with different numbers of assessors. This figure shows the association between increasing the number of assessors and increasing power. The intraclass correlation coefficient (ICC) represents the degree of correlation between different assessors, with a value of zero indicating no more agreement between assessors than would be expected by chance, and a value of one indicating perfect agreement between assessors. The false-positive and false-negative rate were both 20%

Results are shown in Figs. 4 and 5. Results were similar across all false-positive and false-negative rates. Increasing the number of assessors can reduce bias and increase power; however, the extent of this benefit depends on the true ICC; as the ICC increases, the size of the benefit is reduced. For example, for false-positive and false-negative rates of 20%, Fig. 5 shows that when the ICC is 0.10, increasing the number of assessors from one to three will increase the power from 40.1% to 55.9%. However, when the ICC is 0.90, going from one to three assessors only increases the power from 39.6% to 39.7%.

Furthermore, the benefit of adding extra assessors depends on how many assessors there are to start with. For example, for false-positive and false-negative rates of 20%, Fig. 5 shows that increasing the number of assessors from one to three (an increase of two assessors) leads to an increase in power of 15.8% for an ICC of 0.10; however, increasing the number of assessors from five to seven (also an increase of two assessors) leads to a much smaller increase in power of 3.7%.

#### Should we use site or central assessors?

**Fig. 6:**
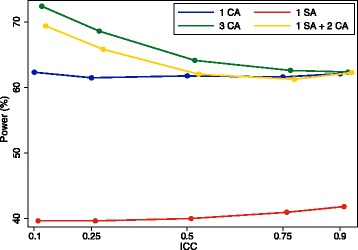
Site vs. central assessors; central assessors have lower false-positive and false-negative rates. This figure shows the association between different adjudication approaches and power. The intraclass correlation coefficient (ICC) represents the degree of correlation between different assessors, with a value of zero indicating no more agreement between assessors than would be expected by chance, and a value of one indicating perfect agreement between assessors. Site assessor: 20% false-positive rate, 20% false-negative rate. Central assessor: 10% false-positive rate, 10% false-negative rate. With three assessors (3 CA, 1 SA + 2 CA), the outcome was adjudicated based on the majority (i.e. if two or more of the assessors adjudicated an event, the outcome was classified as an event, and vice versa). CA, central assessor; SA, site assessor

**Fig. 7:**
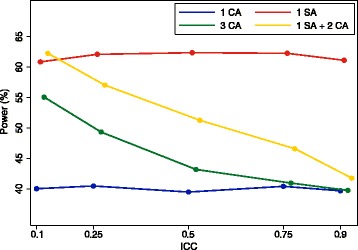
Site vs. central assessors; site assessors have lower false-positive and false-negative rates. This figure shows the association between different adjudication approaches and power. The intraclass correlation coefficient (ICC) represents the degree of correlation between different assessors, with a value of zero indicating no more agreement between assessors than would be expected by chance, and a value of one indicating perfect agreement between assessors. Site assessor: 10% false-positive rate, 10% false-negative rate. Central assessor: 20% false-positive rate, 20% false-negative rate. With three assessors (3 CA, 1 SA + 2 CA), the outcome was adjudicated based on the majority (i.e. if two or more of the assessors adjudicated an event, the outcome was classified as an event, and vice versa). CA, central assessor; SA, site assessor

**Fig. 8:**
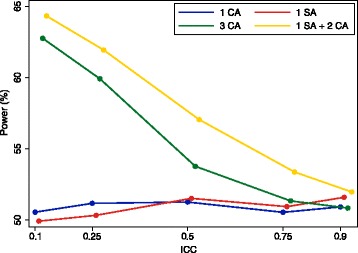
Site vs. central assessors; site assessors have lower false-negative rates, central assessors have lower false-positive rates. This figure shows the association between different adjudication approaches and power. The intraclass correlation coefficient (ICC) represents the degree of correlation between different assessors, with a value of zero indicating no more agreement between assessors than would be expected by chance, and a value of one indicating perfect agreement between assessors. Site assessor: 20% false-positive rate, 10% false-negative rate. Central assessor: 10% false-positive rate, 20% false-negative rate. With three assessors (3 CA, 1 SA + 2 CA), the outcome was adjudicated based on the majority (i.e. if two or more of the assessors adjudicated an event, the outcome was classified as an event, and vice versa). CA, central assessor; SA, site assessor

Results are shown in Figs. 6, 7 and 8. Figure 6 shows results for the scenario where central assessors have lower false-positive and false-negative rates than site assessors. In this scenario, using one site assessor is the worst option. For lower ICCs, using three central assessors is the best option, followed closely by using one site assessor and two central assessors. For very high ICCs, there was little difference between any of the approaches involving central assessors.

Figure 7 shows results for the scenario where site assessors have lower false-positive and false-negative rates than central assessors. In this scenario, using one site assessor is generally the best approach, followed by using one site assessor and two central assessors. For very high ICCs, there was little difference between any of the approaches involving central assessors.

Figure 8 shows results for the scenario where central assessors have lower false-positive rates, and site assessors have lower false-negative rates. Using one site assessor and two central assessors was the best approach across all scenarios, followed by using three central assessors. For very high ICCs, there was little difference between any of the approaches.

#### Should central assessors adjudicate all outcomes, or only suspected events?

**Fig. 9:**
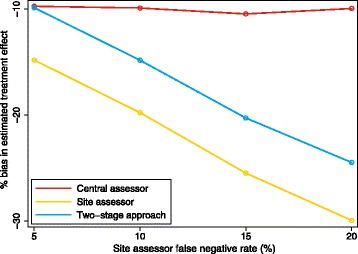
Having central assessors adjudicate suspected events vs. having central or site assessors adjudicate all outcomes. This figure compares the two-stage approach (having site assessors conduct an initial adjudication, and central assessors then adjudicate suspected events) with either having a site or central assessor adjudicate all outcomes. The false-positive and false-negative rates for the central assessor were 5%, and the false-positive rate for the site assessor was 10%. The false-negative rate for the site assessor is shown on the *x*-axis. The intraclass correlation coefficient was set to 0.10

Results are shown in Fig. 9. The two-stage approach (having a central assessor only adjudicate suspected events) was only as good as having a central assessor adjudicate all events when the false-negative rate of the site assessor was very low. When the site assessor’s false-negative rate was high, the two-stage approach led to a large increase in bias compared with having a central assessor adjudicate all outcomes.

#### Independent vs. group adjudication for central assessors

**Fig. 10:**
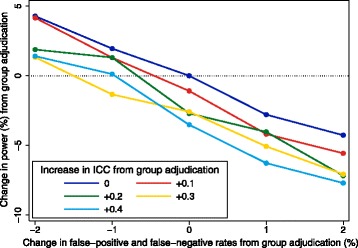
Change in power from group adjudication compared to independent adjudication. This figure shows the change in power from using group adjudication compared with independent adjudication when outcomes are adjudicated by central assessors. The intraclass correlation coefficient (ICC) represents the degree of correlation between different assessors, with a value of zero indicating no more agreement between assessors than would be expected by chance, and a value of one indicating perfect agreement between assessors. The false-positive and false-negative rates for independent adjudication are 20%, and the ICC was 0.50

Results are shown in Fig. 10. Using group adjudication (e.g. an endpoint review committee) was beneficial when it decreased the misclassification rates compared with individual adjudication. Small reductions in the misclassification rate could sometimes be outweighed by very large increases in correlation between assessors, but this was not an issue for larger reductions. Independent adjudication was preferable when group adjudication led to an increase in misclassification rates, or had no impact on misclassification rates but led to an increase in the correlation between assessors.

### Choosing an adjudication approach for clinical trials in ulcerative colitis

As discussed previously, the key factors that will influence the choice of adjudication approach are (i) the comparative misclassification rates of the site and central assessors; (ii) the degree of correlation between assessors and (iii) the additional resource burden associated with central assessment.

#### Comparative misclassification rates between site and central assessors

For trials in ulcerative colitis, there is no gold standard method of adjudication, and it is therefore impossible to estimate the exact misclassification rates of the site and central assessors (as we have no way of determining what the ‘true’ outcome is). However, some published data on the rates of agreement between site and central assessors suggest that the two types of assessor disagree in up to 30% of cases [22, 23]. This is primarily due to site assessors adjudicating higher levels of disease severity than central assessors [22, 23]. It has been suggested that these rates of disagreement are primarily due to misclassification on the part of site assessors, which may be due to their knowledge of patient symptoms [22–24]. For example, if site assessors are aware that the patient is feeling ill, they may subconsciously assume that the endoscopy results must be poor, and may therefore provide a more severe adjudication of disease severity.

#### Degree of correlation between assessors

Published data suggest that there is generally moderate-to-high correlation between central assessors (range 0.50–0.83) [22, 25].

#### Resource burden associated with central assessment

Central assessors would generally adjudicate an endoscopic video through a web portal. The resource burden associated with this is primarily driven by the fact that the endoscopy needs to be videoed at the enrolling clinical site, the video needs to be uploaded into a secure web portal that meets regulatory requirements, a coordinating image management organization must operationalise the review and distribution of images to central assessors, and the central assessors typically need to be paid for their time.

#### Recommendations

Based on the rate of disagreement between site and central assessors, the correlation between assessors, and the resource requirements for central assessment, several adjudication strategies could be proposed. If there is evidence to indicate that the disagreement between site and central assessors is primarily due to misclassification on the part of site assessors, then using only central assessors would be the preferred option. Because the estimated correlation between central assessors is high (>0.50), using more than three assessors would confer little benefit in terms of reducing bias; therefore, using one or three assessors is reasonable. If three assessors were to be used, we would recommend using independent (rather than group) adjudication, as group adjudication would lead to a large increase in resources, without necessarily improving the classification rate.

If we believe that the disagreement between site and central assessors is *not* primarily due to misclassification by the site assessors (e.g. if we believe it was due to misclassification by both site and central assessors) then using a combination approach (one site assessor and two central assessors) may be the preferred option. As before, we would use independent (rather than group) adjudication here.

Regardless of which adjudication approach is chosen, all assessors should be provided training if necessary, and it may be useful to have them adjudicate a number of test cases before the trial starts to ensure adjudication is performed in a consistent manner.

## Discussion

Outcome misclassification can lead to biased treatment effect estimates and reduced power, potentially resulting in an erroneous conclusion regarding treatment efficacy. The implementation of strategies to reduce misclassification is therefore of critical importance in clinical trials. We found that the choice of adjudication approach can have a large impact on trial results, and should be given careful consideration in the planning stages of a clinical trial. We found that no one approach is optimal across all scenarios; instead, the best approach will depend on the specific trial characteristics, notably the misclassification rates of the site and central assessors, and the correlation between assessors. The resource implications of the different approaches is also worth considering.

Our conclusions are summarized in Table 3. Regardless of the adjudication approach chosen, using qualified assessors and providing sufficient training is likely to be of key importance in improving classification rates [3, 15]. For example, in clinical trials of ulcerative colitis assessors could be asked to adjudicate a series of endoscopy videos from a training library prior to starting the trial, to ensure adjudication is being done in an appropriate and consistent way with formal statistical assessment of interrater agreement; furthermore, throughout the trial, further assessments can be conducted to ensure agreement remains within a pre-specified standard with retraining of assessors not meeting those standards. It is also worth noting that in some cases it may be necessary to also provide training for those who are compiling or documenting the evidence that is to be used by the assessor [3]. For example, for imaging outcomes, it may be useful to train those who take the image and to implement quality control processes to ensure that the images are acquired through standardized protocols and are of appropriate quality for adjudication.

**Table 3:** Key points and recommendations

General • No one adjudication approach works best in all clinical trial scenarios. The adjudication approach should be chosen based on the specific trial characteristics, primarily the expected misclassification rates of the site and central assessors, the expected correlation between assessors, and the resource burden associated with different types of assessment. • Regardless of the adjudication approach, trial organizers should ensure that only qualified assessors (i.e. those with adequate experience and expertise) are chosen to perform adjudication • Assessors should be trained before performing adjudication to ensure that consistent approaches are used, particularly in how to deal with complicated cases.
Should we use site or central assessors? • The choice between using site and central assessors should be based on the comparative misclassification rates of the two; whichever type of assessor has the lowest misclassification rates will generally be the better choice (although differences in resource burden between the two approaches should also be considered). When misclassification rates are unknown, as is a common occurrence, a combination approach involving one site assessor and two central assessors may be beneficial.
Should central assessors adjudicate all outcomes, or only suspected events? • Using a two-stage approach where central assessors adjudicate suspected events is typically not a good idea. In situations where central assessors have lower misclassification rates than site assessors, bias will be increased, compared with using a central assessors to adjudicate all outcomes. If a two-stage approach is used, it should be modified so that site assessors pass on all outcomes that could potentially be classified as an event (rather than only outcomes they think *are* events), as this should reduce their false-negative rates, which will improve the performance of the two-stage approach.
How many assessors should we use? • Increasing the number of assessors can reduce bias and increase power, but the size of the benefit depends on the correlation between assessors, as well as how many assessors there are to begin with. In practice, many reported ICCs tend to be >0.50, indicating that there will be rarely much benefit to using more than three assessors (although for trials with ICCs <0.50, using more than three assessors may be beneficial). For outcomes with very high ICCs, using one assessor will typically be sufficient (although using three assessors may still offer some protection against the unlucky scenario in which the one chosen assessor has poor classification rates).
Independent vs. group adjudication for central assessors • For central assessment with multiple assessors, the choice of using either independent adjudication (where each assessors conducts the adjudication independently) or group adjudication (e.g. an endpoint review committee) should be made based on whichever approach is expected to minimize the misclassification rates. However, if group adjudication leads to a very small reduction in the misclassification rate, but is also accompanied by a large increase in the correlation between assessors, then this approach may actually be detrimental. As for other scenarios, the increase in the resource burden should be considered along with the expected increase in classification rates.

## Conclusion

Outcome adjudication is of critical importance to ensure validity of trial results, although no one approach is optimal across all settings. Investigators should choose the best strategy based on the specific characteristics of their trial. Regardless of the adjudication strategy chosen, assessors should be qualified and receive appropriate training.

## Abbreviations

CA: central assessor
ICC: intraclass correlation coefficient
SA: site assessor
